# Tree Species with Photosynthetic Stems Have Greater Nighttime Sap Flux

**DOI:** 10.3389/fpls.2018.00030

**Published:** 2018-01-24

**Authors:** Xia Chen, Jianguo Gao, Ping Zhao, Heather R. McCarthy, Liwei Zhu, Guangyan Ni, Lei Ouyang

**Affiliations:** ^1^Key Laboratory of Vegetation Restoration and Management of Degraded Ecosystems, South China Botanical Garden, Chinese Academy of Sciences, Guangzhou, China; ^2^College of Resources and Environment, University of Chinese Academy of Sciences, Beijing, China; ^3^Guangdong Provincial Key Laboratory of Applied Botany, South China Botanical Garden, Chinese Academy of Sciences, Guangzhou, China; ^4^Department of Microbiology and Plant Biology, University of Oklahoma, Norman, OK, United States

**Keywords:** nighttime sap flow, daytime sap flow, stem corticular photosynthesis, oxygen delivery, water-replenishment

## Abstract

An increasing body of evidence has shown that nighttime sap flux occurs in most plants, but the physiological implications and regulatory mechanism are poorly known. The significance of corticular photosynthesis has received much attention during the last decade, however, the knowledge of the relationship between corticular photosynthesis and nocturnal stem sap flow is limited at present. In this study, we divided seven tree species into two groups according to different photosynthetic capabilities: trees of species with (*Castanopsis hystrix, Michelia macclurei, Eucalyptus citriodora*, and *Eucalyptus grandis* × *urophylla*) and without (*Castanopsis fissa, Schima superba*, and *Acacia auriculiformis*) photosynthetic stems, and the sap flux (*J*_s_) and chlorophyll fluorescence parameters for these species were measured. One-way ANOVA analysis showed that the *F*_v_/*F*_m_ (Maximum photochemical quantum yield of PSII) and Φ_PSII_ (effective photochemical quantum yield of PSII) values were lower in non-photosynthetic stem species compared to photosynthetic stem species. The linear regression analysis showed that *J*_s,d_ (daytime sap flux) and *J*_s,n_ (nighttime sap flux) of non-photosynthetic stem species was 87.7 and 60.9% of the stem photosynthetic species. Furthermore, for a given daytime transpiration water loss, total nighttime sap flux was higher in species with photosynthetic stems (Slope_SMA_ = 2.680) than in non-photosynthetic stems species (Slope_SMA_ = 1.943). These results mean that stem corticular photosynthesis has a possible effect on the nighttime water flow, highlighting the important eco-physiological relationship between nighttime sap flux and corticular photosynthesis.

## Introduction

The significance of nighttime stem sap flow of plants has been discussed in a number of studies in the past decade (e.g., Mancuso and Marras, 2003; Forster, 2014). However, quantifying nocturnal water use is not easy due to technical limitations. For example, the *in situ* measurement of nighttime transpiration (*E*) is difficult and is always overestimated by leaf gas exchange systems (Escalona et al., 2012). The heat dissipation method could simultaneously help us to simply and accurately measure the nighttime sap flow activities and determine the main environmental drivers (Lu et al., 2004; Dawson et al., 2007; Wang et al., 2007; Oishi et al., 2008). Earlier research posited that non-CAM plants close stomata to reduce water depletion at night, meanwhile reducing the absorption of carbon; however, this view caused an underestimate of whole day sap flow (Forster, 2014). Nighttime *E* is strongly influenced by daytime physiological processes, which are attributable to the plant growth rate or the environment (Ludwig et al., 2006). The canopy species of the forest community are shade intolerant and have a higher nighttime to daytime sap flux ratio (*J*_s,n_*/J*_s,d_) (Marks and Lechowicz, 2007). Moreover, previous studies revealed that nighttime sap flux is not only a water recharge for stem water deficit caused by intensive daytime transpiration but also a pathway for oxygen delivery for internal sapwood respiration (Gansert, 2003; Daley and Phillips, 2006; Wittmann and Pfanz, 2014). Consistent with these results, Gansert et al. (2001) found that the xylem sap of was filled with dissolved oxygen when radial influx of oxygen into the sapwood in trees of *Betula pendula* Roth at night. However, the relationship between the nighttime sap flux and oxygen delivery and related mechanisms are poorly investigated and understood.

The responses of plant water flux to environmental variables are also regulated by internal biological traits; for instance, recycling of the internal CO_2_ from the sapwood led to a relative higher whole-tree water use efficiency (WUE) for species that can carry out stem photosynthesis (Nilsen et al., 1993; Vick and Young, 2009). Stems with corticular photosynthesis has no or few stomata in the epidermis, which mainly uses internal CO_2_ for re-assimilation (Wiebe, 1975; Gartner, 1995). And a few carbohydrates produced by corticular photosynthesis are not primarily used for construction purposes but for maintaining and repairing the xylem hydraulic system with the advantage of proximity (Woodruff, 2013; Cernusak and Cheesman, 2015; Bloemen et al., 2016). Schmitz et al. (2012) found that shading the stem of mangrove trees decreased hydraulic conductance and *E*, these results verified the hypotheses that the woody tissue photosynthesis may play a significant role in keeping the balance of the hydraulic transport system.

Based on the perception that there might be a relationship between stem corticular photosynthesis and nighttime water flux, we used TDP (Granier thermal dissipation probes) to measure the nighttime sap flow of seven common tree species with and without photosynthetic tissue in the stem in low subtropical China. The primary goal of this study was to explore whether corticular photosynthesis has an influence on nighttime sap flow. We hypothesized that the nocturnal sap flow of non-photosynthetic stem species would be lower than in photosynthetic stem species, since those species with photosynthetic stems have greater ability to maintain higher hydraulic conductivity and the greater need for oxygen delivery to photosynthetic tissues in the stems.

## Materials and methods

### Site description and examined tree species

The experiment was carried out at three study sites. The first site was located at the Huangmian State Forest Farm, Guangxi Province, China (109°54′ E, 24°46′ N) where *Eucalyptus grandis* × *urophylla* was mainly planted. The research site was 219 m above sea level. Annual precipitation and average temperature were 1750 mm and 19°C, respectively. The *Eucalyptus* plantations mainly contained red and yellow soils. The plantation is routinely managed with phosphorus fertilization. The leaf area index (LAI) of the studied forest during the experiment was 1.92 m^2^ m^−2^, and the stem density was 1,375 stems ha^−1^. We selected five trees for sap flow measurements from the 1st to the 31st of October 2012.

The second site was located at the Heshan National Field Research Station, Chinese Academy of Sciences, Guangdong Province, China (112°54′ E, 22°41′ N). The site elevation was 47 m. This region was dominated by a subtropical monsoon climate, in which annual precipitation and average temperature were 1700 mm and 21.7°C, respectively. This site had reddish soil that was acidic and had low nutrient content (Ren et al., 2007). The forest LAI during the experiment was 2.02 m^2^ m^−2^, and the stem density was 1,019 stems ha^−1^. We selected 3–5 individual trees of the dominant tree species, *Castanopsis fissa, Schima superba, Michelia macclurei*, and *Castanopsis hystrix* to measure the sap flow from the 1st to the 31st of October 2012.

The third research site was located in the South China Botanical Garden (113°22′ E, 23°11′ N), Chinese Academy of Sciences, Guangzhou, China, at an elevation of 49 m. Annual precipitation and average temperature were 1696 mm and 21.9°C, respectively. The canopy tree species were mainly composed of *Acacia auriculiformis* and *Eucalyptus citriodora*. The LAI and the stem density of the forest were 1.97 m^2^ m^−2^ and 804 stems ha^−1^, respectively. The forest soil was acidic and brown. We selected five individual trees for each of *A. auriculiformis* and *E. citriodora*, respectively, the sap flow measurement was also conducted from the 1st to the 31st of October 2013. The characteristics of the studied trees from the three research sites are summarized in Table 1.

**Table 1:** Biometric parameters of the sap flow sample trees from the seven studied species in three sites (Site1: Huangmian State Forest Farm; Site 2: Heshan National Field Research Station; Site 3: South China Botanical Garden).

**Sites**	**Stem corticular photosynthesis**	**Tree species**	**Code**	**Family**	***n***	**DBH (cm)**	**Tree height (m)**	**Sapwood area (cm^2^)**
Site 1	With	*Eucalyptus grandis × Urophylla*	EGU	Myrtaceae	5	9.96 ± 1.08	14.97 ± 6.93	69.24 ± 13.57
Site 2	With	*Castanopsis hystrix*	CH	Fagaceae	3	12.63 ± 0.48	11.08 ± 1.52	94.05 ± 6.57
	Without	*Castanopsis fissa*	CF	Fagaceae	3	21.57 ± 0.74	10.05 ± 4.08	162.88 ± 9.28
	With	*Michelia macclurei*	MM	Magnoliaceae	5	19.54 ± 3.73	12.12 ± 4.04	239.57 ± 81.71
	Without	*Schima superba*	SS	Theaceae	5	13.92 ± 5.32	7.18 ± 1.81	143.43 ± 118.47
Site 3	Without	*Acacia auriculiformis*	AA	Fabaceae	5	30.84 ± 5.49	19.99 ± 1.73	174.21 ± 42.96
	With	*Eucalyptus citriodora*	EC	Myrtaceae	5	30.04 ± 4.66	26.52 ± 3.54	191.34 ± 51.57

*n is the number of trees selected for sap flux measurement. Data are means ± SD*.

All these three sites are located in the low subtropical region of China (at the similar latitude), and characterized by a typical subtropical oceanic monsoon climate. Therefore, the similar climatic and topographic conditions for these three sites, as described above, met the conditions of contrast experiment.

### Environmental monitoring and calculation of vapor pressure deficit

At the Heshan site, we obtained the environmental meteorological data from a meteorological station in the Heshan National Field Research Station, including photosynthetically active radiation (*PAR*, μmol m^−2^ s^−1^), air temperature (*T*, °C), air relative humidity (*RH*, %) and wind speed (m, m s^−1^). At the Huangmian Forest Farm and in South China Botanical Garden, the meteorological data were recorded directly from observation towers (18–23 m) in both forest sites. Photosynthetically active radiation (LI-COR, Lincoln, USA), temperature and humidity (Delta-T Devices Ltd., Cambridge, UK) sensors were mounted on the top of the towers. The vapor pressure deficit (*VPD*, kPa) was calculated according to the following formula (Campbell and Norman, 1998):

$$\begin{array}{l} VPD=a\times \text{exp}\left(\frac{bT}{T+C}\right)\left(1-\text{RH}\right) \end{array}$$

where *a, b*, and *c* are fixed parameters, which were 0.611 kPa, 17.502 (unitless), and 240.97°C, respectively.

As described above, because this study was conducted in three different sites, the way that we collected the environmental data was the best approximation that we could attain in consideration of the equipment available.

### Sap flux measurements

Self-made Granier thermal dissipation probes (TDP) were used to measure sap flow of tress in this study (Granier, 1987). These sensors were consisted of a pair of 20 mm long, 2 mm diameter stainless steel probes, which were vertically inserted into the hydroactive xylem ~10 cm apart. The upper probe was heated by a direct current of 120 mA with a constant power of 0.2 W, whereas the lower probe remained unheated. The Delta-T logger (DL2e, UK) transformed the instantaneous temperature difference of the two probes into a voltage value and collected very 30 s, averaged 10 min (Zhao et al., 2005). Then the sap flow density (g H_2_O m^−2^ s^−1^) was calculated according to the following formula:

$$\begin{array}{l} \text{Js}=119\times {\left(\frac{\Delta T\text{m}-\Delta T}{\Delta T}\right)}^{1.231} \end{array}$$

where Δ*T*_m_ is the temperature difference obtained under zero flow conditions, which was determined separately for each tree over 7–10 days to avoid the underestimation of nocturnal sap flow (Lu et al., 2004). Δ*T* is the instantaneous temperature difference.

### Tree morphological attributes

Considering the possible influences of tree structural factors on nighttime water flux, Pearson's correlation analysis between *J*_s,n_ and *J*_s,n_/*J*_s,d_ and biometric parameters (DBH, tree height, sapwood area) (Table 1) was performed. We chose 14–31 trees of each species in different diameter classes around the plot and used an increment borer to drill out wood core from the stems at breast height. The coloration changes between the heartwood and sapwood helped us to measure Sapwood width.

We determined the wood density and wood water content, as well as the saturated wood water content by following Borchert's method (Borchert, 1994):

Wood density = dry mass/fresh volume

Woody water content = 100 × (fresh mass–dry mass)/fresh mass

Saturated woody water content = 100 × (saturated mass–dry mass)/dry mass.

### Measurement of chlorophyll fluorescence

The stem chlorophyll fluorescence of the seven tree species was determined by a pulse-modulated fluorometer (PAM-2100; Walz, Effeltrich, Germany) to compare the photosynthetic performance among them. We chose three trees from each species to measure chlorophyll fluorescence between 9:00 and 11:00 in the morning. Before measurement, the stems were covered with aluminum foil for 2 h of dark adaptation. Initial fluorescence (*F*_0_) was measured under a weak modulated radiation (0.5 μmol m^−2^ s^−1^), and the dark maximum fluorescence (*F*_m_) was induced by a 0.8-s pulse of saturating light (2,700 μmol m^−2^ s^−1^). The maximal fluorescence in the light (*F*′_m_) was recorded after a second saturating pulse, which is normally lowered with respect to *F*_m_ by non-photochemical quenching. Then the minimum fluorescence of the light-adapted (*F*′_0_) can be measured after a weak far-red light. The variable fluorescence (*F*_v_) was calculated as *F*_v_ = (*F*_m_−*F*_0_). The maximum photochemical quantum yield of PSII (*F*_v_/*F*_m_) for dark adapted stems was calculated according to the following equation: *F*_v_/*F*_m_ = (*F*_m_−*F*_0_)/*F*_m_ (Maxwell and Johnson, 2000), and effective photochemical quantum yield of PSII (Φ_PSII_) was calculated by using the formula as follows: Φ_PSII_ = (*F*′_m_−*F*)/*F*′_m_ (Genty et al., 1989). Besides, the non-photochemical quenching (*NPQ*) was determined according to the equation *NPQ* = (*F*′_m_−*F*′_m_)/*F*′_m_ and the photochemical quenching coefficient (*q*P) was calculated as *q*P = (*F*′_m_ ™ *F*_s_)/(*F*_m_ ™ *F*_m_ ™ *F*_0_) (Maxwell and Johnson, 2000).

### Statistical analysis

A one-way ANOVA followed by a Duncan's test was conducted to test for significant differences (*P* < 0.05) in the chlorophyll fluorescence parameters (*F*_v_/*F*_m_, Φ_PSII_, *NPQ, q*P) among the seven tree species. Pearson correlation analysis was performed to test for significant differences (*P* < 0.05) between nighttime sap flux (*J*_s,n_) and the ratio of nighttime to daytime sap flux (*J*_s,n_/*J*_s,d_) with tree biometric parameters (DBH, tree height, sapwood area) and soil chemical property (soil pH, soil organic matter, total nitrogen, available phosphorous). Independent-sample *t-*test was performed to test for significant differences (*P* < 0.05) in sap flux of photosynthetic stem species and non-photosynthetic stem species for daytime and nighttime. Statistical analyses were performed in SAS 9.2 (SAS Institute, Cary, NC, USA). Figures were plotted using Origin 8.6 (OriginLab Corp., USA). Partial analyses of variations and ANCOVA were evaluated using the Predictive Analytics Software (PASW, IBM, USA). After Ln-transforming variables, the bivariate relationships between daytime and nighttime sap flux were analyzed using standardized major axis (SMA) regression, and the equation parameters was calculated by using SMATR Version 2.0 (Warton et al., 2006).

## Results

### Relationship of *J*_s,n_ with environmental factors and biometric parameters

**Figure 1:**
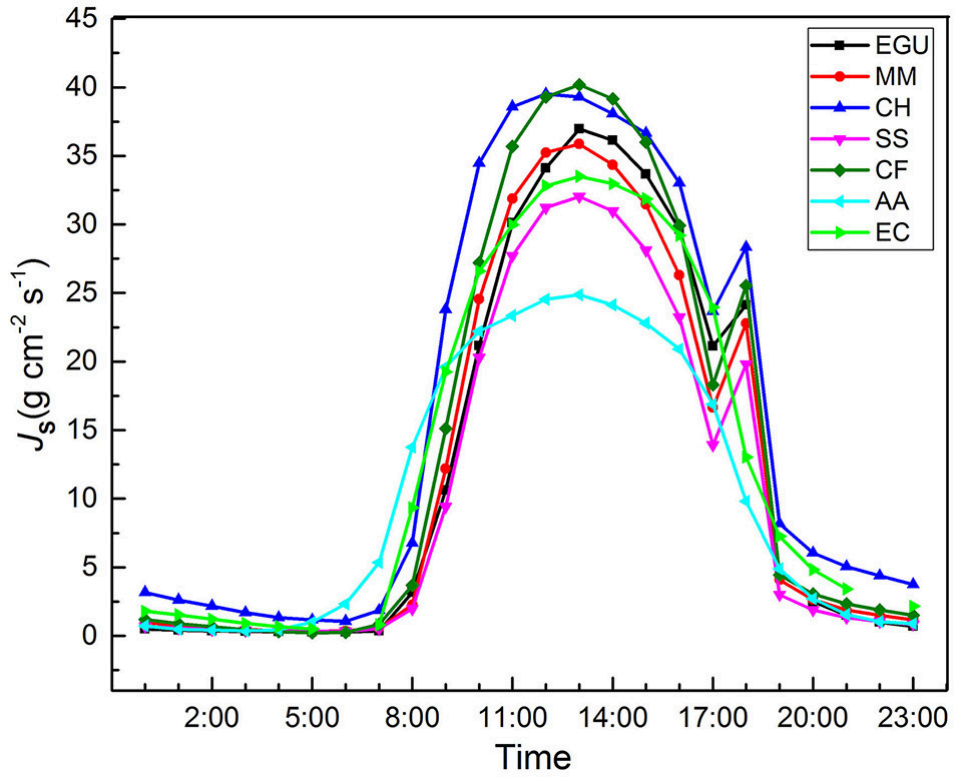
Diurnal patterns of sap flux density in seven studied trees. CH, *Castanopsis hystrix*; MM, *Michelia macclurei*; EC, *Eucalyptus citriodora*; EGU, *Eucalyptus grandis* × *urophylla*; CF, *Castanopsis fissa*; SS, *Schima superba*; AA, *Acacia auriculiformis*. Data point is the average of all days across the study period; bars indicating SD are omitted for clarity.

The daily sap flow pattern of the seven studied trees is shown in (Figure 1). Pearson's correlation analysis between *J*_s,n_ and *J*_s,n_/*J*_s,d_ and biometric parameters (DBH, tree height, sapwood area) showed that the correlation coefficients were very low and non-significant (Table 2), suggesting that canopy position and tree morphological features had little effect on nighttime water flux.

**Table 2:** Pearson correlation between nighttime sap flux (*J*_s,n_) and the ratio of nighttime to daytime sap flux (*J*_s,n_/*J*_s,d_) with tree biometric parameters (DBH, tree height, sapwood area) and soil chemical property (soil pH, soil organic matter, total nitrogen, available phosphorous).

**Variables**	***J***_**s, n**_		***J***_**s, n**_**/*****J***_**s, d**_	
	***r***	***P***	***r***	***P***
DBH (cm)	0.010	0.959	0.270	0.142
Tree height (m)	0.024	0.899	0.155	0.405
Sapwood area (cm^2^)	−0.060	0.748	0.102	0.586
Soil pH	0.161	0.386	0.237	0.199
Soil organic matter (g kg^−1^)	−0.069	0.712	−0.341	0.060
Soil total nitrogen (g kg^−1^)	0.056	0.764	−0.181	0.329
Available phosphorous (mg kg^−1^)	−0.141	0.448	−0.058	0.756

*The quantification of soil nutrient factors is referenced from Lu et al. (2007). Soil chemical properties are for the 0–20 cm soil layer*.

Partial correlation analysis showed a high correlation between *J*_s,n_ and VPD for *E. grandis* × *urophylla* (*P* < 0.000), but there were low correlation coefficients for the other six species, indicating that *J*_s,n_ was mainly for *E* in *E. grandis* × *urophylla*, while it was for water recharge in the other species (Table 3).

**Table 3:** Correlation between daily nighttime sap flux of seven studied species and daily mean vapor pressure deficit (*VPD*).

	**Tree species**	***VPD***	
		***r***	***P***
Photosynthetic stem	CH	−0.143	0.549
	EC	−0.094	0.676
	EGU	0.814	**0.000**
	MM	−0.028	0.898
Non-photosynthetic stem	AA	0.408	0.067
	CF	−0.169	0.431
	SS	0.187	0.417

*Seven tree species were divided into two groups according to the species had corticular photosynthesis or not. Significant effects (P < 0.05) are indicated in bold*.

### Chlorophyll fluorescence of tree stems

**Figure 2:**
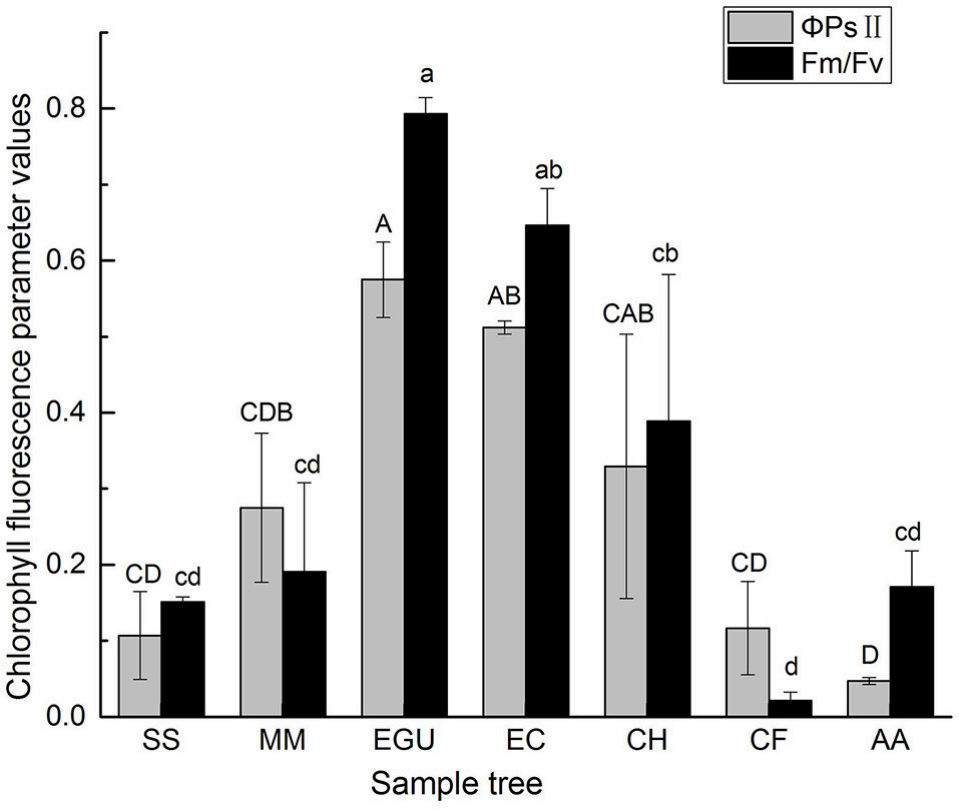
The comparison of chlorophyll fluorescence parameters (*F*_v_/*F*_m_, Φ_PSII_) among the seven studied trees. Data are the mean ± SE (*n* = 3). Letters indicate significant among different species according to Duncan's test; *p* < 0.05 (capital letters for Φ_PSII_ and lowercase letters for *F*_v_/*F*_m_). Species notations same as above.

Light is the energy source driving photosynthesis and the signal that regulates plant growth and development. The stems of tree species with chlorophyll can carry out stem photosynthesis (Supplementary Figure 1). Two *Eucalyptus* species had corticular green tissue with a thickness of ca. 2 mm, and *C. hystrix* and *M. macclurei* also had green bark, but its thickness was ca. 0.5 mm. In contrast, *C. fissa, S. superb*, and *A. auriculiformis* had no distinct green tissue on the trunk. To evaluate the different photosynthetic capacities of the stems, chlorophyll fluorescence parameters were measured in the sample trees of the seven species. The results showed that *F*_v_ /*F*_m_ (Maximum photochemical quantum yield of PSII) and the Φ_PSII_ (effective photochemical quantum yield of PSII) values were lower in non-stem photosynthetic species (*C. fissa, S. superb*, and *A. auriculiformis*) compared to stem photosynthetic species (*C. hystrix, M. macclurei, E. citriodora*, and *E. grandis* × *urophylla*; Figure 2).

As shown in Table 4, there was a significant difference in the *F*_v_ /*F*_m_ value and the Φ_PSII_ value in the sampled trees. However, no significant difference in *q*P and *NPQ* among the sampled trees was observed (Table 4).

**Table 4:** Variance analysis of the chlorophyll fluorescence parameters *F*_v_*/F*_m_ (Maximum photochemical quantum yield of PSII), Φ_PSII_ (effective photochemical quantum yield of PSII), *q*P (photochemical quenching), and *q*N (Non-photochemical quenching) of stem among seven studied species.

**Variable**	**Source**	**Sum of squares**	***df***	**Mean square**	***F*-value**	***P*-value**
*F*_v_/*F*_m_	Model	1.469	6	0.245	7.510	0.001
	Error	0.457	14	0.033		
	Corrected total	1.926	20			
Φ_PSII_	Model	0.761	6	0.127	5.970	0.003
	Error	0.297	14	0.021		
	Corrected total	1.058	20			
*q*P	Model	0.449	6	0.075	0.600	0.725
	Error	1.738	14	0.124		
	Corrected total	2.187	20			
*q*N	Model	0.396	6	0.066	0.58	0.744
	Error	1.606	14	0.115		
	Corrected total	2.002	20			

### Pattern of nighttime sap flow and daytime sap flow for species with and without photosynthetic stems

**Figure 3:**
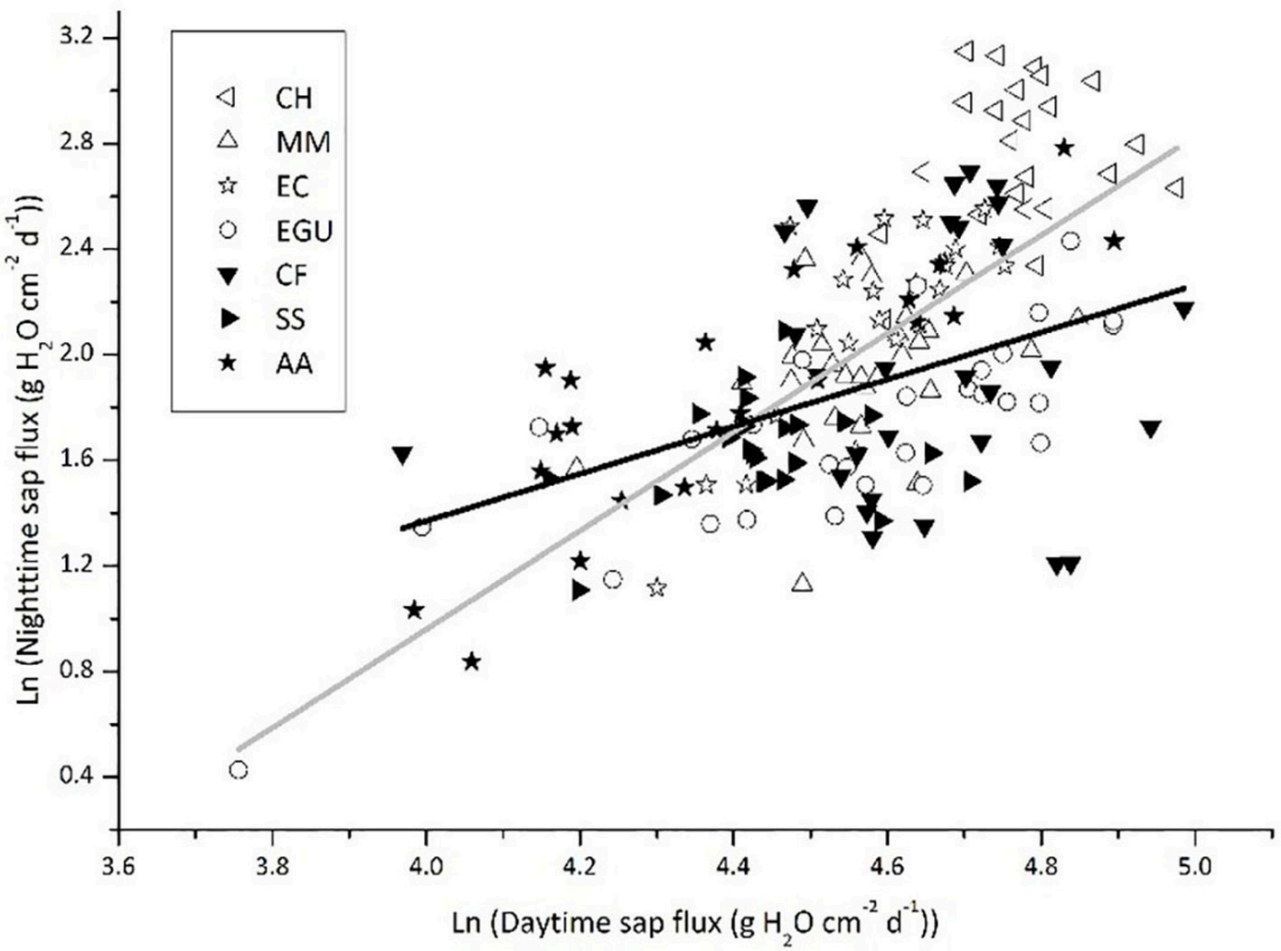
Nighttime sap flux as a function of daytime sap flux in trees with or without stem photosynthesis. The sap flux values reported for each species are the means of 3–5 trees. The solid gray line represents the standardized major axis regression across the four species with stem photosynthesis: y = 2.680x – 10.261 (*r*^2^ = 0.485, *P* < 0.0001); the solid black line represents the standardized major axis regression across the three species without stem photosynthesis: y = 1.943x – 6.936 (*r*^2^ = 0.211, *P* < 0.0001).

We plotted *J*_s,n_ as a function of *J*_s,d_ for the two groups of species, namely those with photosynthetic stems (*C. hystrix, M. macclurei, E. citriodora*, and *E. grandis* × *urophylla*) and without photosynthetic stem (*C. fissa, S. superb*, and *A. auriculiformis*). As shown in Figure 3, the standardized major axis slope of the photosynthetic stem group (Slope_SMA_ = 2.680) was higher than that of the non-photosynthesis stem group (Slope_SMA_ = 1.943; *P* = 0.008), indicating that the *J*_s,n_ of the trees with stem corticular photosynthesis was more active. After removing the data of *E. grandis* × *urophylla*, where *J*_s,n_ was mainly for *E* as demonstrated by the correlation analysis in Table 3, the slope of the other three photosynthetic stem species (Slope_SMA_ = 3.244, *r*^2^ = 0.543) was even higher than that of the non-photosynthetic stem trees (*P* = 0.001). If the data were not Ln-transformed and plotted by OLS regression, the slope of species with stem corticular photosynthesis (Slope_OLS_ = 0.167, *r*^2^ = 0.364, *P* < 0.0001) would still be significantly higher than that of non-photosynthetic stem species (Slope_OLS_ = 0.071, *r*^2^ = 0.198, *P* < 0.0001; *P* < 0.001). We have also conducted ANCOVA analyses and found that the group effect (stem corticular photosynthetic capability) was significant if *J*_s,d_ is incorporated (Table 5).

**Table 5:** ANCOVA results of the effects of group, *J*_s,d_ (daytime sap flux) or the interaction of group and *J*_s,d_ on *J*_s,n_ (nighttime sap flux).

**Source**	**Type III Sum of squares**	***df***	**Mean squares**	***F***	**Significance**
Corrected model	18.925	3	6.308	44.849	0.000
Intercept	6.547	1	6.547	46.548	0.000
Group	1.597	1	1.597	11.356	0.001
*J*_s,d_	13.707	1	13.707	97.450	0.000
Group × *J*_s,d_	1.704	1	1.704	12.113	0.001
Error	24.052	171	0.141		
Total	734.485	175			
Corrected total	42.977	174			

*Seven tree species were divided into two groups according to the species had corticular photosynthesis or not*.

As shown in **Figure 5**, there was a significant difference in *J*_s,d_ (*P* = 0.013) and *J*_s,n_ (*P* < 0.000) between the two groups of tree species, both *J*_s,d_ and *J*_s,n_ were higher in stem photosynthetic species than in non-stem photosynthetic species.

## Discussion

**Figure 4:**
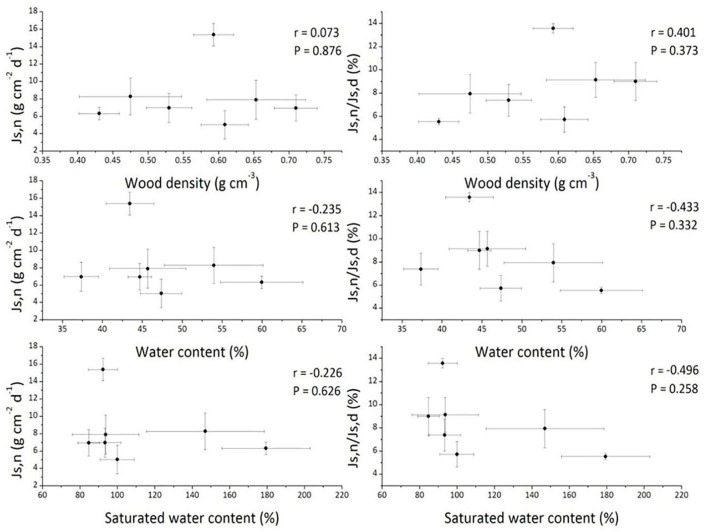
The nighttime sap flux (*J*_s,n_) and the ratio of nighttime to daytime sap flux (*J*_s,n_/*J*_s,d_) vs. wood density, wood water content, and saturated wood water content across the seven studied tree species. Data are means ± *SD*.

We investigated the possible influences of tree morphological attributes (DBH, tree height, sapwood area), meteorological environmental factors and soil environmental factors on the nighttime sap flow in this study. Wood density may exert an influence on sap flow due to its high correlation with hydraulic conductance and carbon assimilation (Santiago et al., 2004; Pratt et al., 2007). For example, De Dios et al. (2013) reported that nighttime stomatal conductance (*g*_s_) was negatively correlated with wood density in *E. tereticornis*. In our study, there were no significant correlations between *J*_s,n_ or *J*_s,n_/*J*_s,d_ and wood density, wood water content, or saturated wood water content (Figure 4), indicating that nighttime water flux was not influenced by wood type among these seven tree species. Furthermore, the effects of soil chemical properties on *J*_s,n_ were also quantified, and the results showed that neither *J*_s,n_ nor *J*_s,n_/*J*_s,d_ were affected by soil acid alkalinity, soil organic matter or available phosphorous (Table 2). Consistent with these results, the research on *Arabidopsis thaliana* also indicated that nighttime water flux had no relationship with soil nutrient conditions (Christman et al., 2009). Interestingly, nearly every soil nutrient indicator was negatively correlated with *J*_s,n_ (Table 2), suggesting that nighttime *J*_s_ may be indirectly used for nutrient uptake, which was consistent with the observations by Scholz et al. (2007). However, the decreased nighttime *J*_s_ could not be attributed to soil nutrient status due to a negative relationship between wood density and *J*_s,d_ that was observed simultaneously (*r* = −0.709, *P* = 0.074, data not shown). All results demonstrated that tree form features and soil factors did not contribute to variation in nighttime sap flow in this study (Table 2). Although the possible effects of soil condition and meteorological factors on sap flow were discussed as above, it is noteworthy that the imbalance of the selection of seven tree species in three sites cannot be ignored, which caused the precondition of detecting sites effects is not available in this study.

Even though the trunk surface has low radiation transmittance, stems containing green tissue can use the limited energy source of radiation for effective carbon assimilation (Saveyn et al., 2010; Wittmann and Pfanz, 2016). Due to morphological features (for example, the absence of stoma or similar organelles) and weak photosynthesis, it is difficult to apply a gas exchange method that is based on the infrared CO_2_-absorption principle for the *in situ* measurement of stem photosynthesis (Teskey et al., 2008; Zhu et al., 2012). Therefore, as an efficient and non-destructive method, the *in vivo* measurement of chlorophyll fluorescence has been widely used in plants and is considered an appropriate way to evaluate stem photosynthesis (Wittmann and Pfanz, 2016). An increase in *F*_v_/*F*_m_ and Φ_PSII_ reflects higher light use efficiency in plants (Baker and Rosenqvist, 2004; Baker, 2008). The results of the our study showed that there were lower values of *F*_v_/*F*_m_ and Φ_PSII_ in the species without stem green tissue compared to those with stem green tissue (Figure 2), demonstrating that stem photosynthetic species had a higher utilization of the weak light energy.

**Figure 5:**
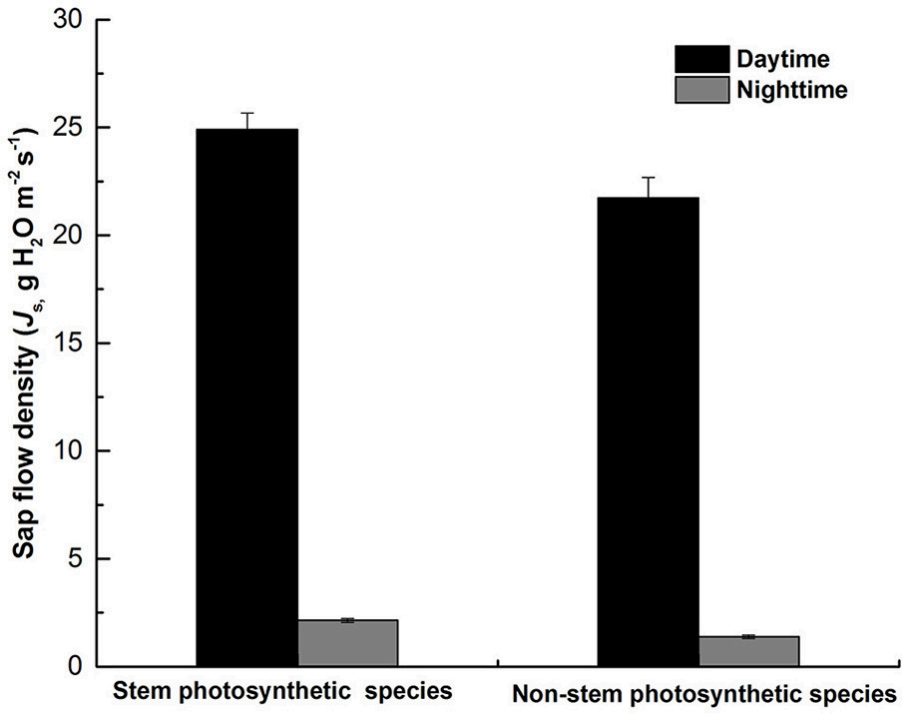
The difference of daytime mean sap flux (*J*_s,d_) and nighttime mean sap flux (*J*_s,n_) between the two groups of species (stem photosynthetic species and non-stem photosynthetic species). Data are means ± SE.

In our study, we found that species with chlorophyll in the stem have a more active *J*_s,n_ (Figure 3). On the one hand, species that have photosynthetic stems can use the weak light to produce carbohydrates during daytime. The accumulation of photosynthates helps the stem maintain a high metabolic rate at night, which depends on nocturnal sap flux to deliver oxygen. Similar results have also been reported by Del Hierro et al. (2002), which indicated that the higher *J*_s,n_ in the species have stem photosynthetic capacity is probably favorable for oxygen transport to xylem or sapwood parenchyma. In addition, the research on bark chlorophyll content and photosynthesis reported that photosynthesis and respiration were higher in the tree species with more chlorophyll in the stem (Ren et al., 2009). And highly positive correlation was found between branch photosynthesis and respiration in nine tree species (Berveiller et al., 2007). Tree species with stem photosynthesis can use internal stem CO_2_ to produce extra O_2_ during the day (Pfanz et al., 2002; Borisjuk and Rolletschek, 2009). Nevertheless, the stem green tissue is not “oxygen source” but “oxygen sink” due to that the light is unavailable at night. Despite the respiration during the night is not as intensive as that during the daytime because of the lower nocturnal temperature, the respiration of green tissue is always higher than that of xylem living cells, which probably leads to hypoxia (Butler and Landsberg, 1981; Pallardy, 2008). Thus, the O_2_ conveyed by nighttime sap flux can alleviate hypoxia stress to some extent (Eklund, 2000; Gansert, 2003; Mancuso and Marras, 2003; Sorz and Hietz, 2006, 2008). On the other hand, the carbohydrates produced by stem corticular photosynthesis may have a role in maintaining and repairing the xylem hydraulic system (Steppe et al., 2015; Bloemen et al., 2016). Then, a high transpiration rate during the day can lead to an intensive stem water deficit, thus requiring more water recharge during the following night (Fuentes et al., 2013). We found that *J*_s,d_ and *J*_s,n_ of non-photosynthetic stem species were lower (*P* = 0.013 and *P* < 0.000, respectively; Figure 5) than photosynthetic stem species. Similarly, Schmitz et al. (2012) found that the hydraulic conductivity was lower in the non-photosynthetic stem, demonstrating that the stem corticular photosynthesis has a significant role in regulating water transport. Based on the above analysis, our study provided observation data explaining that corticular photosynthesis has an effect on the nighttime water flow.

## Conclusion

Nocturnal water flux plays a role in delivering oxygen to the internal parenchymatous tissues of the stem xylem wood and in replenishing water consumed by transpiration during day. In our study, we found that species with stem corticular photosynthesis are likely to have higher *J*_s,n_, which is largely consistent with our hypothesis that the nocturnal sap flow of non-photosynthetic stem species would be lower than in photosynthetic stem species. These prospective result will be verified by the improvement of experimental scheme, and we suggest that it is time to rethink the relationship between corticular photosynthesis and nocturnal water flux. However, due to the existence of species-specific effects, additional studies should be done to fully assess the influence of stem photosynthesis on nighttime water flux, one of the issues that is worth paying attention to is the possible chemical composition variation in the cortex below the bark and the dilution effect from sap flow. In addition, the quantification of oxygen concentration in the sapwood needs to be investigated in the future studies.

## Author contributions

XC and JG: Conducted the experiments, analyzed the experimental data, and wrote the manuscript; JG and PZ: Designed the experiment. PZ, HM, LZ, GN and LO: Contributed to revising manuscript.

### Conflict of interest statement

The authors declare that the research was conducted in the absence of any commercial or financial relationships that could be construed as a potential conflict of interest.

## Supplementary material

Supplementary Figure 1 The seven studied tree species with stem photosynthesis: *Castanopsis hystrix* **(a)**, *Michelia macclurei* **(b)**, *Eucalyptus citriodora* **(c)**, *Eucalyptus grandis* × *urophylla* **(d)**; and without stem photosynthesis: *Castanopsis fissa* **(e)**, *Schima superba* **(f)**, and *Acacia auriculiformis* **(g)**.
