# Supplementary figures and images for: Tree Species with Photosynthetic Stems Have Greater Nighttime Sap Flux
Source: Front Plant Sci. 2018 Jan 24;9:30. doi: 10.3389/fpls.2018.00030 (PMC5787579; doi:10.3389/fpls.2018.00030)

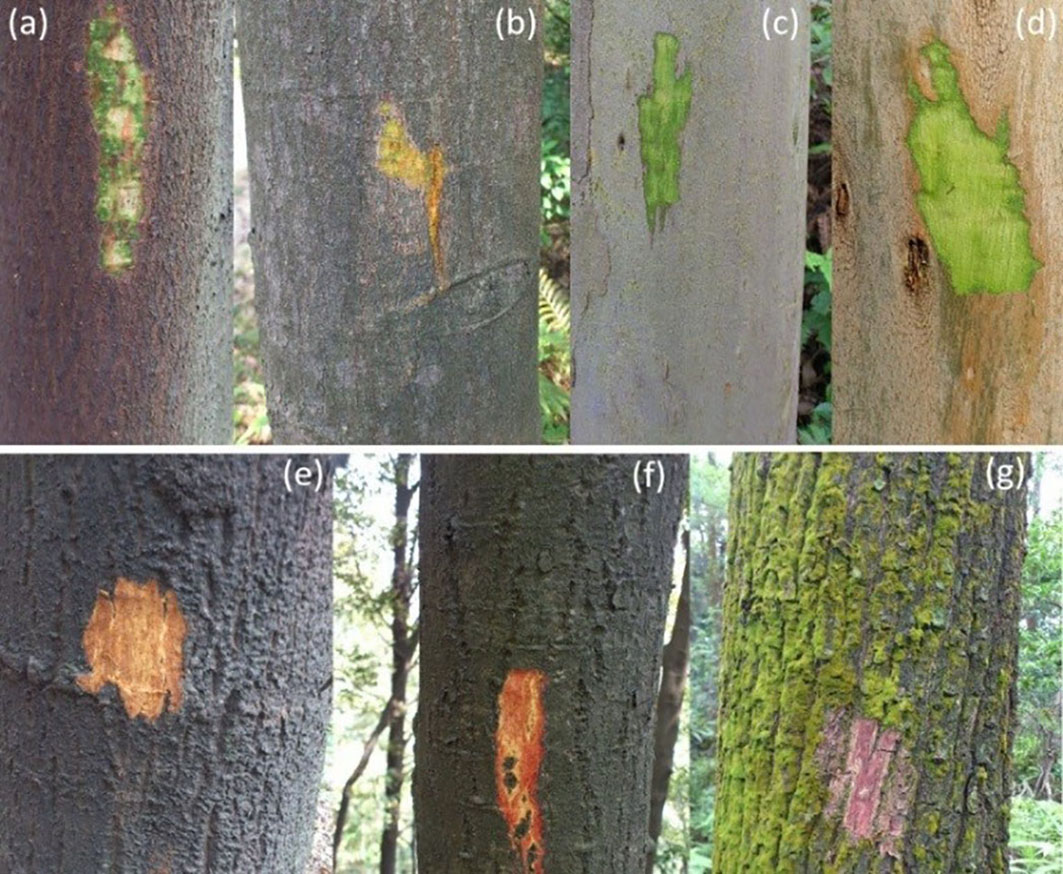

Supplement: Supplementary Figure 1 — The seven studied tree species with stem photosynthesis: Castanopsis hystrix (a), Michelia macclurei (b), Eucalyptus citriodora (c), Eucalyptus grandis × urophylla (d); and without stem photosynthesis: Castanopsis fissa (e), Schima superba (f), and Acacia auriculiformis (g). [file Image1.JPEG]
